# Exploring parents’ attitudes towards a multicentre cohort study of children with burns injuries: A qualitative interview study

**DOI:** 10.1177/20595131221098526

**Published:** 2022-06-30

**Authors:** Philippa Tollow, Nicola Marie Stock, Diana Harcourt

**Affiliations:** Centre for Appearance Research, 14262 University of the West of England, Bristol, UK

**Keywords:** Burns, scalds, public involvement, cohort, qualitative, paediatric, thematic analysis

## Abstract

**Background:**

Burn injuries affect more than 60,000 children every year in the UK, with many experiencing scarring as a result. Scarring can be highly variable, and research is required to explore the factors that may influence variability, as well as the psychosocial impact of these injuries on children and their caregivers. A multicentre burns cohort study is being planned to investigate genetic determinants of scarring and long-term psychosocial outcomes. Public involvement (PI) is an essential element of the design and feasibility stages of this planning. As part of this work, this study aimed to gain an in-depth understanding of parents’ attitudes towards participation in burns research, specifically a longitudinal cohort study of children with small burns (<10% total body surface area [TBSA]).

**Methods:**

In total, 16 parents of children with burns took part in semi-structured interviews regarding their experiences of taking part in research and their attitudes towards the potential future cohort study. Interviews were audio-recorded, transcribed verbatim and analysed using Reflexive Thematic Analysis.

**Results:**

Four themes were identified: ‘Acknowledging trauma’; ‘Aligning research with experience’; ‘Research as a reciprocal relationship’; and ‘Contributing to change’.

**Discussion:**

These four themes represent factors that parents suggested were important for acceptability, relevance, recruitment and retention of participants into a longitudinal multicentre cohort study of children with a burn injury and their caregivers.

**Conclusion:**

The findings of this study will be incorporated into the design of such a study, as well as having wide reaching relevance for research in the field of paediatric burn injuries.

**Lay Summary:**

*Background to this subject*

More than 60,000 children experience a burn injury every year in the UK and many of these injuries lead to scarring. We know that the extent of this scarring can vary, and we know that some children and their parents/caregivers manage well but others struggle with the challenges they face after having a burn. Researchers would like to carry out research on these topics, including asking participants to take part in research over several years to find out how genetics might influence scarring, as well as their psychological experiences over this time. Before they conduct this study, it is very important that researchers understand parents' attitudes towards this kind of research. The current study aimed to find out parents' opinions and ask what issues were important to them when taking part in burns research.

*Details of how the work was conducted*

Parents of children who had experienced a scald (a type of burn injury) were asked to take part in a research interview. In total, 16 parents took part in this study. We recorded these interviews and analysed them, looking for patterns and shared experiences in participants' interviews.

*What we did and did not learn from this study*

We found four themes in the interview data: ‘Acknowledging trauma', ‘Aligning research with experience', ‘Research as a reciprocal relationship', and ‘Contributing to change'. Overall, these themes suggest that parents were mostly supportive of a ‘burns cohort study’, but they have also highlighted some important considerations for this research and other future burns research studies.

## Introduction

Burn injuries are estimated to affect approximately 64,000 children in the UK every year, with an estimated 6639 of these children requiring hospital admission. The majority of these children are under the age of five years at the time of injury, representing approximately 75% of those who attend hospital with a burn injury.^1^ The most common cause of these injuries is scalds (e.g. hot drink spills, hot bath water),^2^ and while many paediatric burn injuries are minor and do not require hospital admission, the impact of such injuries is wide ranging and may include a significant physical and psychosocial impact. Many children will experience scarring as a result of their burn injury, and we know that greater scarring is likely to result from large burns, wounds that take a long time to heal and wounds requiring multiple surgical procedures.^3^ However, scarring can be highly variable and further research is required to explore the factors that may influence such scar variability. In addition, research suggests that emotional adjustment to scarring is also highly variable and many children and families require further psychosocial support.^4^ Significantly more research is needed to understand the long-term psychosocial outcomes of children with a burn injury and their parents, since there are few longitudinal studies of psychosocial wellbeing in this area.^5^ Further research in each of these areas would allow for both medical and psychological support to be tailored to the individual and allow for the provision of better quality care.

In order to address these gaps in the literature, researchers plan to conduct a multicentre burns cohort study, investigating genetic determinants of scarring and long-term psychosocial outcomes of burn injuries in children and their caregivers. Their planned study will build upon previous research exploring the genetic determinants of scarring in adults who have sustained burn injuries^6,7^ and provide vital insight into the experiences of children and their caregivers in the months and years following injury. While many studies have investigated health-related quality of life in children who have had a burn injury, few studies have explored this longitudinally, and particularly in small scalds or children under the age of five years.^5^ This study would recruit children aged under five years who have sustained a small area scald, and their parents or caregivers, since predictors of parental wellbeing is another under-researched area. A scald, in this study, is defined as a form of burn injury that has been caused by hot liquid or steam.

An important stage of the initial planning for such a research project is public involvement (PI), including working closely with patients and members of the public to ensure that the research is focusing on relevant research questions, using appropriate methodology, and is acceptable to the population that it seeks to recruit. PI is increasingly being recognised as a vital element of research, with potential benefits including increased research validity, greater recruitment and retention into research, and more meaningful outcomes.^8^ PI was identified as an important element of early planning for a burns cohort study, particularly as many parents of children with burn injuries will have experienced this as a traumatic event. Previous research has highlighted the impact of feelings of guilt and shame on parents of a child with a burn injury,^9^ as well as the relationship between these feelings and psychological adjustment^10^; therefore, approaching parents in the right manner and at the right time is an important consideration for all research in this area. In addition, research including genetics is widely believed to be ethically sensitive and potentially daunting for participants; therefore, it is important that parents’ views on this element of the study are considered and incorporated throughout. Situating PI as a key component of the early design and planning stages of research, is a valuable way of ensuring future research is informed by the perspectives of the population that it seeks to recruit and/or benefit from the outset.

An example of similar PI work can be seen in researchers’ exploration of factors that might contribute to participation in a gene bank to improve understanding of cleft lip and/or palate (CLP).^11^ This ‘cleft gene bank’ would aim to recruit a cohort of individuals born with a cleft, and collect genetic, social and demographic data in order to investigate the connections between genetic and environmental factors in cleft aetiology (as well as a range of additional research questions). Using qualitative interviews and focus groups, the study by Williams et al. sought to identify factors that might motivate parents of children with CLP to participate in such research, as well preferences regarding the practicalities of being approached to take part. This work allowed the researchers to identify positive attitudes towards a cleft gene bank in many parents who took part, but also to identify sensitive issues that required consideration in their future research methodology (e.g. reassuring parents of research integrity). Similarly, existing research is able to offer some insight into individuals’ reasons for declining to participate in research more broadly,^12^ participants’ retrospective experiences of taking part in research^13^ and their attitudes towards genetic research more broadly^14–16^; however, we are not aware of any published paper that reports similar PI work in the burns population.

As described above, researchers in the UK are currently in the planning and development phases of a multicentre burns cohort study, to investigate genetic determinants of scarring and long-term psychosocial outcomes of burn injuries in children and their caregivers. As part of PI work for the proposed cohort study, we aimed to gain an in-depth understanding of parents’ attitudes regarding a longitudinal cohort study of children with burns, including the acceptability of this type of research and the factors that would have encouraged or prevented them from taking part in a study like this when their child had their burn injury. It is hoped this will provide valuable insight into parents’ attitudes towards their child taking part in research after a burn injury, with implications for both the proposed cohort study and the field of burns research more broadly.

## Methods

### Design

This was a qualitative study, with semi-structured interviews conducted via telephone or video call depending on each participant's preference. All interviews were audio-recorded and transcribed verbatim. Participants were recruited from an existing pool of individuals who had taken part in previous research regarding children's burn injuries and had expressed an interest in taking part in future studies. Inclusion criteria were based on the provisional design for a ‘burns cohort study’ and the following was required of the participants: a parent of a child who has previously sustained a burn injury (when the child was aged <5 years); aged ≥18 years; and able to provide informed consent and take part in a research interview conducted in English. This research received a favourable ethical opinion from the University of the West of England Research Ethic Committee (approval number: HAS.20.11.052).

### Participants

A total of 16 participants took part in this study (15 mothers, 1 father), including one parent dyad. All participants had a child who had a scald injury when aged under five years. The mean age of participants was 35.5 years (age range = 24–46 years) and the mean age of the child at the time of scald injury was two years (range = 9 months–4 years).^1^ See Table 1 for further demographic details.

**Table 1.** Participant demographic information.

		Frequency (n = 16)
Sex	Female	15 (94)
	Male	1 (6)
Interview mode	Video call	5 (31)
	Telephone call	11 (69)
Ethnicity	White British	13 (81)
	Other White	1 (6)
	Pakistani	1 (6)
	Asian Indian	1 (6)

Values are given as n (%).

Interviews lasted for an average of 38 min, with a total interview duration of 9 h 37 min across 15 interviews.

### Data collection

Parents who were eligible to participate in this study were identified from an existing database of individuals interested in taking part in future research (as described above) and contacted via phone or email, depending on the preference specified when taking part in previous research. This represented approximately 80 individuals who were approached and given information about the research and invited to contact the researchers if they were interested in taking part. All of those who subsequently expressed an interest in participation were given an information sheet, privacy notice and consent form. If they wished to proceed then they were asked to provide written consent via the consent form and complete a short demographic questionnaire, before arranging a time for the interview to take place. All participants received an online shopping voucher in return for their participation.

Interviews were conducted by the first author and guided by an interview schedule designed by the researchers. This interview schedule was based on the researchers’ experience conducting research in this area, previous experience conducting PI work and the aims of this research study. The interview schedule included initial questions relating to the participant and their child's experience of treatment and support relating to the burn. The researcher then gave the participant further information about the proposed ‘burns cohort study’, followed by questions relating to the parents’ attitudes towards these research topics, appropriate timing to approach parents about research, barriers and facilitators to taking part in research, and their attitudes towards specific elements of the proposed study design (e.g. frequency of follow-up). While this interview schedule acted as a guide, questions were added, removed or asked in an alternative order depending on appropriateness and the responses of the participant.

### Data analysis

Interview data were analysed by the first author, using reflexive thematic analysis and conducted from a critical realist approach. Working from this approach meant that the researchers acknowledge the subjective nature of these parents’ experiences, while also assuming the existence of an ‘external reality’ in order to facilitate practical application of the research. The first author is an experienced qualitative researcher, whose previous research has primarily focused on using qualitative methods to explore the experiences of people with appearance-altering conditions, as well as experience conducting research with parents of children who have had a burn injury. While the nature of qualitative research means that the author's prior knowledge and experiences inevitably influence data collection and analysis, efforts were made to focus the analysis closely on the data collected and to reflect on any assumptions made during the research process. The analysis process was informed by the work of Braun and Clarke and followed six steps: ‘dataset familiarisation’; ‘data coding’; ‘initial theme generation’; ‘theme development and review’; ‘theme refining, defining and naming’; and ‘writing up’.^17–19^ Computer Aided Qualitative Data Analysis (CAQDAS) software was used to aid analysis. Pseudonyms have been used throughout reporting of the results and any potentially identifiable information has been removed. Standards for Reporting Qualitative Research (SRQR) reporting guidelines have been used in reporting.

## Results

Four themes were identified through analysis: ‘Acknowledging trauma’’; ‘Aligning research with experience’; ‘Research as a reciprocal relationship’; and ‘Contributing to change’. All four themes are presented in detail below (see Table 2 for a summary of themes and demonstrative quotes).

**Table 2.** Themes, with exemplar quotes.

Theme	Participant quote
Acknowledging trauma	‘It's always really important to acknowledge how this is a difficult time’
Aligning research with experience	‘Her feelings towards her burn have changed, and that's not been captured anywhere’
Research as a reciprocal relationship	‘It sort of felt like someone was checking up on you in a way, and making sure that you weren't alone.’
Contributing to change	‘If it helps in the future, then it's worth it. If it helps another family what we went through, then yeah, it's definitely worth it.’

### Acknowledging trauma

Many participants discussed taking part in research through the lens of the initial trauma that they experienced when their child had their burn injury. They stressed the importance of researchers in this area acknowledging the trauma that they had experienced and suggested that an empathetic approach should be incorporated throughout the design process and in interaction with participants. They discussed the importance of researchers being sensitive in the timing of the first approach to discuss the research, ensuring that this was not at a time of high distress for the parents or the child, and highlighting the point of discharge as a potential time point to consider first approaching parents to take part in a cohort study.

‘*It just has to be done in the most sort of sensitive way*’ *– Laura*

Parents also stressed the importance of research and researchers adopting a non-judgemental approach throughout, particularly in relation to the guilt that many parents experience with relation to their child's burn, and ensuring that this was carefully considered throughout recruitment, data collection, analysis and dissemination.

‘*It's always really important to acknowledge how this is a difficult time*’ *- Helen*

Several parents described their child as their priority above all else after their burn injury and suggested they would only have considered taking part in research when reassured that their child was safe and recovering. They emphasised the importance of judging this timing carefully, as not to discourage parents from taking part in research. Parents also suggested that any information about research should be first given verbally, before being followed up with written information, as the trauma associated with the burn meant that it was hard to process additional information around this time.

‘*You've got so many things to process, and [research is] not your top priority at the moment*’ *– Laura*

### Aligning research with experience

Participants’ discussion of many elements of the research design—including frequency, duration and method of data collection—were focused on alignment between the research and their experience of having a child with a burn injury. They discussed their expectation that frequency of participation (e.g. how often they were asked to complete outcome measures [questionnaires]) would be proportionate to the severity of the burn, the impact on daily life and the rate of change.

‘*Maybe like initially for the first year maybe every three months, and then after the year I’d probably say like every six months for a couple of years. And then [the effects of the burn] would probably, after a couple of years, it would start settling anyway, and it's not going to really change that much more.*’ *– Victoria*

More severe burns, a greater impact on daily life and a faster rate of change of the wound/scar were all expected to justify more frequent data collection, whereas parents would expect that participation would be less frequent when the burn was less severe, having less impact on daily life or changing less rapidly. In line with this, parents suggested that they would have felt happy to take part in research frequently during the first-year post burn, and then in decreasing frequency in subsequent years. Although burns specialists had often indicated to parents that the rate of observable change would slow after two years, many participants suggested that they continued to see change after this time, and particularly social/psychological changes as their child went to school or became more aware of their burn scar. Several parents suggested that research would be a valuable way to capture these changes, as they did not feel they were being recorded elsewhere.

‘*Her feelings towards her burn have changed, and that's not been captured anywhere*’ *– Liz*

Many suggested that they would be willing to take part in research for many years post burn, but that they would expect to opt out when the research felt less relevant for their experience, and for researchers to include this opt-out check-in at each point of the research. When discussing their willingness to participate over a long period, many parents reflected on the long-term impact that the burn injury had on the lives of them and their family, and the relevance that the topic would continue to have for them.

‘*I would be happy to continue to do it, because I don’t think there comes a time in your life as a parent where you just forget that that happened.*’ *– Samantha*

### Research as a reciprocal relationship

Many parents described the importance of ensuring that research participants felt valued and satisfied with the process of taking part in research, and that the experience of participation as a whole should be a positive one. A thread running through many of these conversations was the reciprocal nature of the relationship between participant and researcher, with both benefitting from the exchange. For example, many stressed the importance for participants of including support information with all research materials, including signposting to psychological support where appropriate. This was highlighted by several participants as an opportunity to inform parents of support services that they may not be aware of. Parents described discovering support services through chance encounters or study materials and suggested that research could be a useful avenue for dissemination of these resources:

‘*And I thought, “Oh, why isn't anyone signposting us to this?” Perhaps at the end of the form if you could have the links to some charities or some support.*’ *– Laura*

Many parents also suggested that the opportunity to discuss their feelings and reflect on their experiences was a benefit of taking part in research, as well as feeling supported in an otherwise potentially isolating experience.

‘*I believe that it's always actually helping to talk about it. So being able to talk about it and to talk about how you feel in that moment, it does help a lot.*’ *– Rachel*

In particular, taking part in research with repeated participation time points was seen to represent a positive experience and a feeling of being looked after.

‘*It was quite nice as well, because [taking part in research] sort of felt like someone was checking up on you in a way, and making sure that you weren't alone.*’ *– Victoria*

Participants also discussed the importance of regular feedback when taking part in research and suggested they would appreciate receiving detailed updates on research progress, including recruitment numbers, response rates, interim findings, achievements of the research and impact.

‘*What am I doing? Like, why am I taking the time to do this? Sometimes it's hard to understand how it can help, or how it does help, so it would have been good to feed back*’ *– Gemma*

Many parents suggested that these elements would be crucial to retaining participants over a period of time, as well as asking participants for their feedback on the experience of taking part and any changes that should be made during the research process (e.g. feedback on recruitment). While many participants discussed being motivated to take part in order to help others in similar situations (see the theme ‘Contributing to change’), they also suggested that financial incentives to take part made them feel valued, felt like a fair exchange for their time and often allowed them to buy something for their child. Within each of these elements, there was a feeling of reciprocity between researcher and participant.

### Contributing to change

A theme present across all participants’ accounts was their altruistic motivation to take part in research. Often, they described the trauma and difficulties that they and their child had experienced in relation to the burn injury and discussed wanting to help others in similar situations. They discussed taking part in research as having the potential for others to receive better treatments, more support and potentially have better scarring outcomes, and this being a strong motivating factor in their own participation.

‘*If it helps in the future, then it's worth it. If it helps another family what we went through, then yeah, it's definitely worth it.*’ *– Karen*

This was particularly salient to many participants due to the otherwise overwhelmingly negative nature of the experience of their child's injury, and the opportunity for taking part in research was seen to be a ‘positive outcome’ to have come out of an otherwise traumatic experience.

‘*I’m very happy to take part in the trials and things because any positive outcome I can find out of what has happened I would be keen to be part of*’ *– Lucy*

Further strengthening these feelings was the value that participants saw in particular research studies, with many expressing an interest in the proposed topics of research for the burns cohort study. Many parents suggested that scarring was an important outcome for them, and they wished to understand more about the factors that influenced this, with several parents comparing their child's burn scar to other family members’ scars and considering the potential for genetic components to influence these outcomes.

‘*If anything we can find out to… to help scarring better, or to help people with burns to not have scars, then that’s… that’s something like going forward, isn’t it?*’ *– Rachel*

Parents also identified parental adjustment and psychological support as crucial elements of their experience, with many suggesting that further research on this topic would be valuable in order to understand the experiences of parents and how this changes over the course of the years after a burn injury.

‘*I think it's also important for research to completely understand how parents feel, because everyone will feel differently about it and some people will recover quicker than others.*’ *– Samantha*

## Discussion

This study aimed to gain an in-depth understanding of parents’ attitudes regarding a proposed longitudinal cohort study of children with scalds; including the acceptability of this type of research to parents of a child with a burn injury and the factors that would have encouraged or prevented them from taking part when their child had a burn injury. The following four themes were identified that contribute towards this aim: ‘Acknowledging trauma’; ‘Aligning research with experience’; ‘Research as a reciprocal relationship’; and ‘Contributing to change’. With discussion of child and parental experiences of a burn injury woven through each of these themes, these findings could be said to reflect previous research reporting on experiences of burn injuries, while also providing additional insight into how these experiences might influence parents' attitudes towards burns research. Overall, these findings suggest that parents were supportive of a ‘burns cohort study’, but they have also highlighted some important considerations for this research, as well as future research recruiting children with burn injuries and their parents.

The findings of this study are broadly in line with previous studies exploring participants’ experiences of taking part in research. Such studies have highlighted the importance of clear participant information, discussion with trusted professionals and feedback of results,^13^ as well as the importance of trust when conducting health research.^20^ While participants in this study were considering these issues in relation to a future study, many of these elements can be seen reflected in this study's findings. The findings of this study also echo those found by Williams et al. when exploring parents’ attitudes towards a cleft gene bank.^11^ In both studies, parents highlighted the value that they placed in good quality research, wanting to take part to help others and the importance of supporting research participants throughout the process. Parents in both the cleft and burns groups also stressed the importance of providing clear information to participants and incorporating sensitivity into recruitment strategies. The complex nature of burn injuries and the trauma often associated with burns in children led to a particular emphasis on the latter, with parents in this study keen to stress the importance of acknowledging the traumatic experience that often led to their contact with burn services, the guilt surrounding their experiences as parents of a child with a burn and changes in their experiences over time.

The themes presented in this paper have important implications for conducting research regarding paediatric burn injuries, and participants drew a clear link between these factors and recruitment and retention in future studies. For example, suggesting that ‘acknowledging trauma’ during the recruitment process would play an important role in whether they would have chosen to take part, or that the research design (e.g. frequency of participation) should align with their experience of the burn injury. Many of these elements will require careful consideration when implementing them in future research design, for example, it may be difficult to ensure within one research study that the frequency of data collection feels appropriate for all participants. However, it is hoped that understanding of these conceptual and pragmatic elements will aid in the design of sensitive and appropriate research methodologies, and ultimately increase retention in a longitudinal study of children with burns. Importantly, these findings could also be said to echo studies exploring reasons that individuals decline invitations to part in research,^12^ and this would be a helpful topic to explore more explicitly going forward, for example, in a pilot study ahead of a multicentre cohort study. Drawing on the findings of this study and the themes presented above, specific recommendations for future research are presented in Table 3 (it should be noted that these are key recommendations and are not intended to be an exhaustive list).

**Table 3.** Recommendations for conducting longitudinal research, with children with burn injuries and their parents, based on the themes generated by the current study.

Key recommendations	Related theme
Recruitment should take a non-judgmental empathetic approach, ensuring that parents are not asked to take part during a time of distress (e.g. for inpatients this could be just before discharge).	Acknowledging trauma
Information about the study should first be provided in both verbal and written format, before giving the participant time to make a decision about taking part.	Acknowledging trauma
Frequency of participation in research should echo patient/parent experiences of the burn injury, with more frequent participation in the year following the injury when more rapid change is anticipated and less frequent participation in following years.	Aligning research with experience
The option to opt out of the study at each follow-up should be made clear to participants at each time point.	Aligning research with experience
Public involvement should be embedded throughout the research cycle in order to ensure that research aims, objectives and design is in line with patient's experiences.	Aligning research with experience
Research participation should include personal contact with the researcher(s) and parents should be given regular feedback and study updates throughout participation, with a particular emphasis on the positive contribution of their participation.	Research as a reciprocal relationship
All research materials should include information about sources of relevant support, including psychological support, for children with a burn injury and their parents.	Research as a reciprocal relationship
Research should consider the use of incentives (e.g. shopping vouchers) in order to ensure that participants feel valued and appropriately rewarded for their time.	Research as a reciprocal relationship
Research aims and objectives should be set in collaboration with individuals with lived experience, in order to ensure that these are felt to represent a positive contribution to improving patient experiences and/or outcomes.	Contributing to change

### Methodological considerations

An important consideration when interpreting the findings of this study is that recruitment was from a sample of individuals who had previously taken part in research and expressed an interest in hearing about future studies. While this does not allow us an insight into the attitudes of parents who would be reluctant to take part in future research, this is not believed to detract from the findings. Rather, the findings of this study should be interpreted within the understanding that these themes represent attitudes towards a ‘burns cohort study’ from parents who have previously expressed an interest in taking part in research. This provides an important insight into the factors that would influence their future participation, particularly as previous research in which this group took part did not include a genetic component, and will allow the researchers to use these findings as a basis for the initial study design. However, this also highlights the importance of conducting a pilot study before proceeding with the main study, in order to explore the number of individuals visiting a hospital burns service who would be willing to consent to this type of research and exploring reasons for non-consent, as well as piloting the collection, storage and analysis of genetic materials.

There are also several groups with whom it would be beneficial to conduct further research or PI work and who were not included in the present study. First, it should be noted that the participants who took part in this study were predominantly mothers and therefore we cannot be sure that these findings reflect the views of fathers of children with a burn injury, although no differences were noted between the views of one father and the 15 mothers who took part in this study. Similarly, this study was not able to capture the perspective of parents who do not speak English. This is a particularly important area of future study, to understand the views of this population and the specific barriers that they may experience to taking part in this kind of research. Finally, this research focused on parents of children aged under five years, as this is the age at which participants were planned to be recruited into a burns cohort study. However, future research would also benefit from exploring children's attitudes to taking part in research relating to burns, particularly as it is likely that children would also be asked to complete longitudinal outcome measures as part of a burns cohort study as they reached an appropriate age.

## Conclusion

This study aimed to gain an in-depth understanding of parents’ attitudes regarding a proposed longitudinal cohort study of children with burns. The findings suggest a number of factors that should be incorporated into the study design to ensure acceptability, relevance and sensitivity to parents of children with burns, and provide an important insight into the experiences of taking part in research after a burns injury, which are applicable to a wide range of burns research studies.

## Supplemental Material

sj-docx-1-sbh-10.1177_20595131221098526 - Supplemental material for Exploring parents’ attitudes towards a multicentre cohort study of children with burns injuries: A qualitative interview study Click here for additional data file. Supplemental material, sj-docx-1-sbh-10.1177_20595131221098526 for Exploring parents’ attitudes towards a multicentre cohort study of children with burns injuries: A qualitative interview study by Philippa Tollow, Nicola Marie Stock and Diana Harcourt in Scars, Burns & Healing

## Notes

1. These details were reported by participants and not sourced from medical records, therefore may be subject to recollection error.
