# Supplementary material for: Exploring parents’ attitudes towards a multicentre cohort study of children with burns injuries: A qualitative interview study
Source: Scars Burn Heal. 2022 Jun 30;8:20595131221098526. doi: 10.1177/20595131221098526 (PMC9253984; doi:10.1177/20595131221098526)
Supplement: sj-docx-1-sbh-10.1177_20595131221098526 - Supplemental material for Exploring parents’ attitudes towards a multicentre cohort study of children with burns injuries: A qualitative interview study [file sj-docx-1-sbh-10.1177_20595131221098526.docx]

**Exploring parents’ attitudes towards a multi-centre cohort study of children with burn injuries: a qualitative interview study: Interview schedule**

**[Questions are in bold text,** Prompts are in Roman text]

*Questions in this schedule will act as a guide; however, questions may be added or removed depending on appropriateness and the responses of the participant.*

*Introductory questions*

- **To start with, could you tell me what interested you in talking to me today?** How did you find out about this research? What interested you about taking part?
- **Thank you, that’s really helpful. As you know, this interview is part of a study to find out what people think about the possibility of setting up a ‘burn's cohort research study’. We’ll talk more about that research in a while, but I wonder if first you could start by telling me a bit more about you and your child’s experience of a burn?** Perhaps you could start by telling me a bit about what happened when your child was burnt? When did the burn happen? What treatment did your child receive? Are they currently having any treatment? What kind of support have they/you received? What kind of support are you receiving now?
- *<Explain details of proposed research and answer any questions that the participant has in order to clarify their understanding>*
- **What are your first thoughts when you hear about this research?** How do you feel about research to find out more about scarring? How do you feel about research to find out more about how people’s emotional outcomes after a burn injury? Do you think this is useful?

*Approaching parents to take part*

- **With this type of study, researchers at the hospital would want to approach parents quite quickly after the burn injury to invite them to take part in the research. How do you think you would have felt about that?** When do you think you would have wanted to be approached about a study like this? Is there a bad time to be approached about research like this? Do you think you would have wanted to hear the information at an appointment at the hospital or would you like to receive written information first? Who would you feel comfortable approaching you about research like this – doctor, nurse, research nurse not involved in care, someone else?

*Data collection*

- **As part of this research, researchers would ask parents and children to provide different types of information. Some of this would be related to genetics and one of the things they would want to do would be to collect some saliva from the child who had the burn, in order to record information about the child’s genetics and investigate how genetics influence scarring over time. How do you think you would have felt about that?** When do you think would be an okay time to ask a child to do this? Would you have had any concerns about this? Do you think your child would have been happy to do this after they had their burn?
- **Similarly, I wonder how you would have felt if researchers had asked for a blood sample from your child?** When would have been an okay time to ask a child to do this? Would you have had any concerns about this? Do you think your child would have been happy to do this after they had their burn?
- **Another type of information that would be collected as part of this research is how children and parents are feeling and adjusting after the burn. This would usually be through questionnaires completed by the parents or carers. How do you think you would have felt about that?** When would have been an okay time to ask you to do this? Would you have had any concerns about filling in these types of questionnaires? How would you feel about answering questions about your own wellbeing, as well as your child’s wellbeing? Is there anything you wouldn’t have wanted to be asked about?

*Follow-up*

- **As part of this potential cohort study, researchers are planning to collect more information over a period of time to see how things change for children and parents/carers over months or years, for example, with questionnaires sent to those who are taking part. The researchers haven’t decided yet how long this would be or how often they would ask parents/carers or children to do this. What do you think about this?** How long after your child’s burn do you think you’d have been willing to take part in this type of research for? How often after your child’s burn do you think you’d have been willing to answer these types of questionnaires? How would you feel about attending the hospital with your child for extra appointments if this was part of the research? What do you think would encourage you to keep taking part over time? What do you think would put you off from keeping taking part over time?

*Facilitators and barriers to taking part*

- **What do you think would encourage people to take part in this type of research?** Can you think of anything that would have encouraged you to take part? Sometimes people are given a thank you for their time, like a shopping voucher, how would you feel about that?
- **What do you think would stop people from taking part in this type of research?** Can you think of anything that would have stopped you from taking part in this type of research?

*Closing questions*

- **Thank you so much for talking to me today, that’s all the questions that I have. Is there anything else you would like to add?** Do you have any questions for me?
